# Theoretical Model of Bending Moment for Straight Mortise-and-Tenon Joints with Wooden Pegs Involving a Gap

**DOI:** 10.3390/ma15051835

**Published:** 2022-03-01

**Authors:** Bin Hu, Jian Cai, Chun Yang

**Affiliations:** 1 School of Civil Engineering and Transportation, South China University of Technology, Guangzhou 510641, China; 201710101210@mail.scut.edu.cn (B.H.); cvjcai@scut.edu.cn (J.C.); 2 College of Architecture & Engineering, Guizhou Minzu University, Guiyang 550025, China; 3 State Key Laboratory of Subtropical Building Science, South China University of Technology, Guangzhou 510641, China

**Keywords:** straight mortise-and-tenon joint, mechanical modeling, low-cycle reversed loading test, bending moment, gap

## Abstract

The stress mechanism of a straight mortise-and-tenon joint with wooden pegs in traditional residential wooden structures was analyzed, and a theoretical moment-rotation model of the joint was derived. To verify the model, three full-scale joint specimens were fabricated and subjected to low-cycle reversed loading tests. All specimens showed tensile cracking parallel to the grain at the top or bottom of the tenon neck. The theoretical calculation results are consistent with the experimental results. The results of the parametric analysis based on the theoretical model show the following: the rotational stiffness and bending moment of the joint increase as the beam width increases; as the beam height increases, the moment increases, but the initial stiffness of the joint is only slightly impacted; as the column diameter increases, the initial stiffness and moment increase, and the free rotation of the joint decreases; as the gap between the mortise and tenon increases, the initial stiffness and moment decrease; as the sliding friction coefficient increases, both the rotational stiffness and moment of the joint increase, and the increase is greater after the joint yields than before.

## 1. Introduction

Mortise-and-tenon joints (MTJs) are often used in traditional residential wooden structures to transfer internal forces and dissipate energy among the members. Research [1] has shown that the seismic energy dissipated by MTJs under earthquakes can be as high as approximately 30% of the total energy. Traditional residential wooden structures, which have better seismic resistance, energy-saving and corrosion resistance than reinforced concrete structures [2,3,4,5], not only have use value, but also ornamental value with strong national characteristics, appreciated by tourists. However, due to long-term exposure to the external environment, a large number of existing traditional dwellings suffer different degrees of damage, which urgently needs to be repaired. As the core area of the force transmission, the damage to MTJs could cause the structure to collapse. Therefore, the MTJs play an important role in guaranteeing the safety of traditional dwellings. At the same time, research on the mechanical behaviors of MTJs is also the foundation of the protection and repair of traditional residential wooden structures.

**Figure 1:**
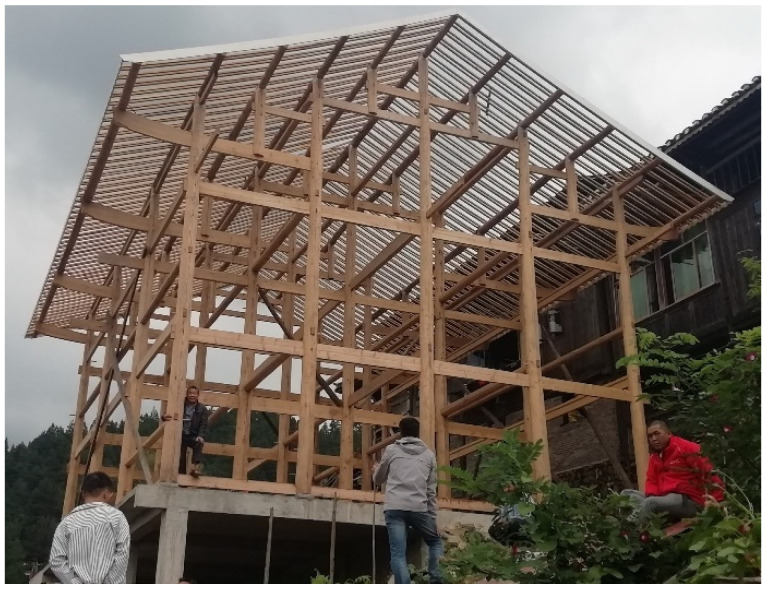
Structure of traditional dwellings in Southeast Guizhou.

In recent years, numerous studies of the mechanical behaviors of MTJs have been conducted by many domestic and foreign scholars. Some have focused on the rotational behavior of the MTJs without degradation. Xie et al. [6] investigated the seismic performance of straight mortise-and-tenon joints (SMTJs) of different forms by conducting low-cycle reversed loading tests, analyzed the influence of different conditions, including joint types, model scales, and lengths of tenon and wood types, on the seismic behavior of SMTJs. and obtained the seismic performance indexes of different forms of SMTJ (such as moment-rotation hysteretic curve, backbone curve, rotational stiffness and its degradation law, etc.). The results indicated that the failure patterns were mainly squeeze deformation of mortise and tenon, and partial pulling out of the tenon. William et al. [7] studied the mechanical performance of four types of wood-pegged MTJs and proposed a mechanical calculation model considering the influence of wooden pegs. Feio et al. [8] investigated the behavior of a traditional mortise and tenon timber joint using physical testing of full-scale specimens and drew a conclusion that the parameters that affect the ultimate load of the timber joint mostly are the compressive strength of wood perpendicular to the grain. Pang et al. [9] investigated the moment-carrying capacity of the traditional post-beam joints via static loading experiments, and described the differences of moment resistance, joint stiffness, and failure modes with or without the beam shoulder. The results indicated that the beam shoulder had a great effect on the performance of the joint. Li et al. [10] conducted low-cycle loading experiments with four mortise-tenon wooden frames and obtained the failure modes and hysteretic performance of the MTJs. Xie et al. [11] analyzed the stress mechanism of an SMTJ and theoretically derived its moment-rotation calculation equation. Based on these theoretical formulas, the effects of parameters (such as beam height, beam width and friction coefficient, etc.) on the rotational moment of unidirectional SMTJs were analyzed. Pan et al. [12] derived a mechanical model for the moment-rotation relationship of the SMTJ that considers the influence of the pull-tenon capacity of the joint and drew a conclusion that the contribution of different compression regions to flexural stiffness is different. The effects of different types of damage on joint mechanical performance have also been analyzed by some scholars. Pan et al. [13] established a numerical model verified with the experimental data of through-tenon joints. The influences of joint gap, transverse elastic modulus of timber, and length of the large tenon on the bending capacity of the joint were analyzed. Based on the results of stress analysis, a three-fold multi-parameter *M*-*θ* mechanical model was proposed. This mechanical model can be verified with the test results and applied to the stress analysis of timber frames. Xie et al. [14] experimentally studied the rotational behavior of degraded Chinese traditional MTJs with different degradation types and different degradation degrees and proposed a hysteretic model for degraded traditional MTJs which was in good agreement with the experimental results. Chang et al. [15] in Taiwan studied the rotational performance of traditional Nuki joints with gaps and developed a theoretical model to estimate the rotational stiffness of the joints. The results showed that the butted Nuki joints had significantly lower initial stiffness and bending moment than the continuous Nuki joints, and the butted Nuki joints were more sensitive to the gaps, which leads to a large initial rotation angle. Xue et al. [16] analyzed the stress mechanism of through-tenon joints in ancient buildings and derived the theoretical moment-rotation equation that considers different degrees of looseness of the joint, and the theoretical calculation results were in good agreement with the experimental values. He [17] derived a theoretical moment model for loose through-tenon joints that considers the influences of looseness and asymmetry on their stress state. The results indicated that the proposed model was consistent with the experimental results and that the theoretical model reflected joint mechanical behavior. Meanwhile, the results also showed that stiffness varied with the development of parameters such as gaps, deformation, and contact area. Ogawa et al. [18] derived a method of theoretical estimation with a gap as a parameter for the mechanical performance of MTJs, conducted experiments to validate the theory, and numerically analyzed the influence of the size of the gap on mechanical properties (rotational stiffness, initial slip and strain energy). The results indicated that the size of the gap had a significant effect on the mechanical performance of the joints, indicating the importance of taking the gap into consideration in evaluating the mechanical performance of the joints. From previous research results on MTJs, it was found that there is little research on the MTJs of traditional residential wooden structures in Southeast Guizhou, as shown in Figure 1. Southeast Guizhou, China, where a large number of ethnic minority villages are located, is characterized by a large number of traditional dwellings with wooden structures. The main material used in these structures is Chinese fir (Cunninghamia lanceolata). The structural system generally consists of four three-span structures with eleven purlins, nine columns and two penetrations, and its side joints are mainly SMTJs with wooden pegs (SMTJs/WPs). At present, no scholars have conducted theoretical research on the mechanical performance of the SMTJs/WPs involving a gap, which is caused by wood shrinkage and could significantly affect the mechanical performance of the joints. Since the theory is far behind the actual needs of the project, this restricts research on the mechanical properties of traditional residential wooden structures to a certain extent. Therefore, it is necessary to explore the theoretical calculation method of moment-rotation of SMTJs/WPs in traditional residential wooden structures.

In this study, theoretical analyses for the mechanical performances of the SMTJ/WP involving a gap were performed under cyclic loading. The judgement criteria for different working states were given and the bending moment of the joint at each working state was obtained. A theoretical moment model of this type of joint verified via a full-scale experiment was proposed and a parametric analysis was carried out on this basis, which provides support for research on traditional dwellings in Southeast Guizhou.

## 2. Stress Mechanism of SMTJs/WPs in Traditional Wooden Structure Dwellings

### 2.1. Construction of the SMTJ/WP

**Figure 2:**
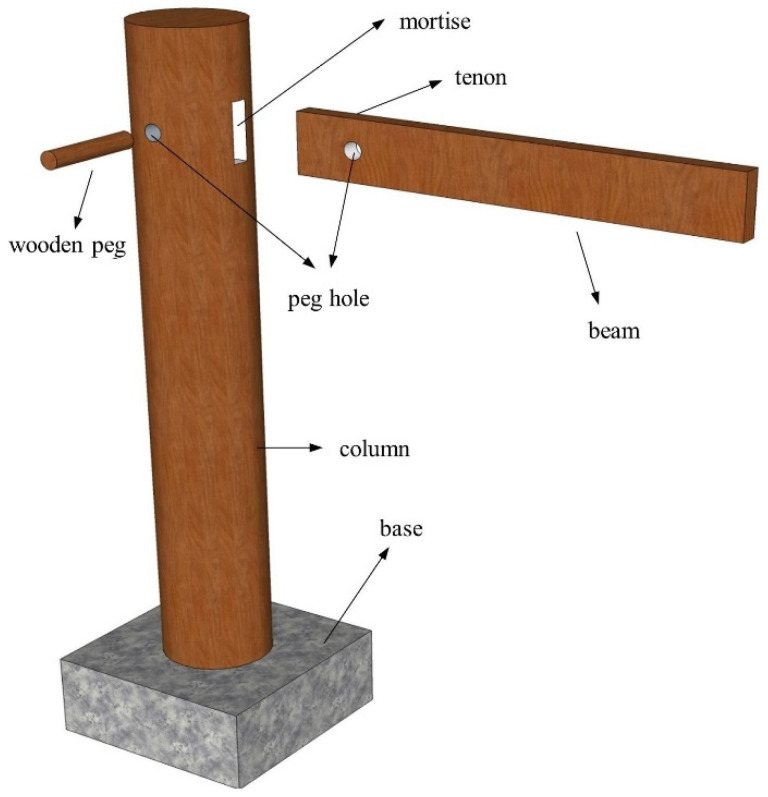
Diagram of SMTJ/WP.

The SMTJ/WP is a special joint consisting of a tenon, a mortise, and a wooden peg, with no adhesive involved and the wooden peg inserted in the middle of the joint to lock the tenon, as shown in Figure 2. In contrast to the case of an SMTJ without a wooden peg, the tenon is inserted into the mortise and then locked with the wooden peg, which can effectively prevent the tenon from being pulled and damaged and thus guarantee the structural integrity. When a structure is subjected to a horizontal load, the SMTJ/WP can withstand a certain bending moment and produce a certain rotation, which is similar to that of MTJs without wooden pegs [14,19,20]; thus, it consists of a semirigid joint between the rigid and hinged connections.

### 2.2. Stress Mechanism of the SMTJ/WP

**Figure 3:**
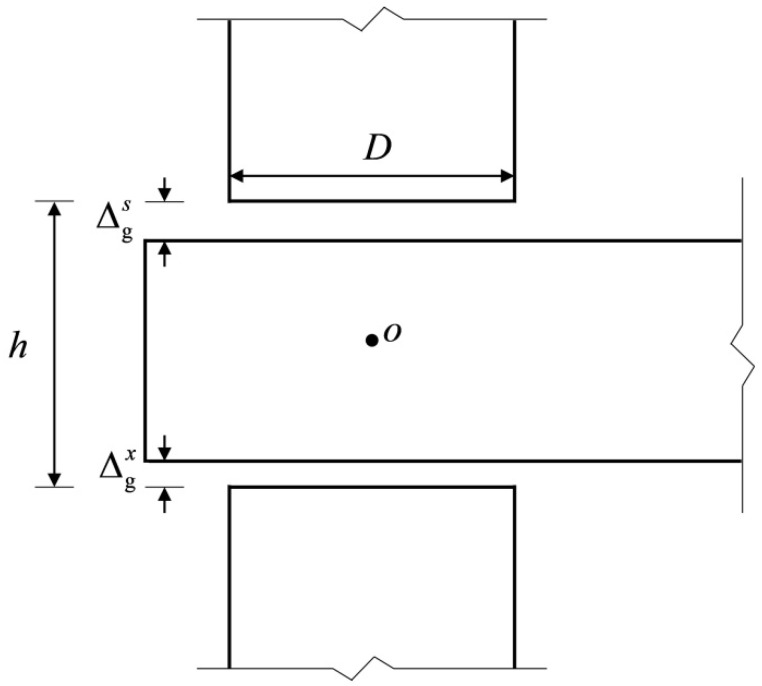
The detailed dimensions of the SMTJ/WP. Note: ${∆}_{g}^{s}$ and ${∆}_{g}^{x}$ are the gaps between the upper and lower surfaces of the tenon and the mortise, respectively; *D* is the diameter of the column; *h* is the height of the mortise.

There is a gap between the tenon and the mortise due to the shrinkage of the tenon wood [21] (Figure 3). Because the relative sizes of ${∆}_{g}^{s}$ and ${∆}_{g}^{x}\;$ can influence the stress state of the tenon, the stress mechanism of the joint is analyzed by assuming ${∆}_{g}^{s}>{∆}_{g}^{x}.$ (Note that the case of ${∆}_{g}^{s}<{∆}_{g}^{x}$ is similar to the case of ${∆}_{g}^{s}>{∆}_{g}^{x}$, and the case of ${∆}_{g\;}^{s}={∆}_{g}^{x}$ can be simply merged into the case of ${∆}_{g}^{s}>{∆}_{g}^{x}$ in terms of the stress state).

**Figure 4:**
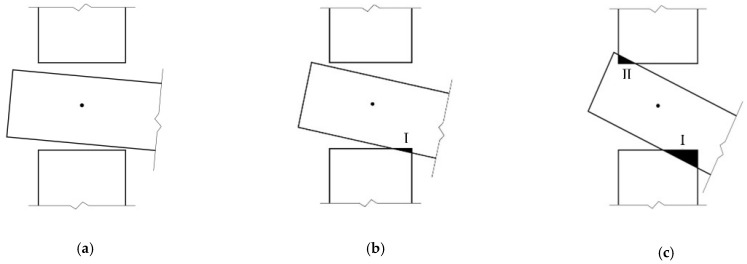
Mechanical analysis of SMTJ/WP: (**a**) free rotation; (**b**) Area I entering compression state; (**c**) Area I and Aea II entering compression state.

When subjected to an external load, the tenon first rotates freely in the mortise (as shown in Figure 4a). As the rotation increases, the tenon overcomes the gap between its lower surface and the mortise and then contacts the mortise. As the load increases, the lower surface of the tenon and the mortise compress against each other, so the tenon is radially compressed perpendicular to the grain, and the element with the mortise is compressed parallel to the grain (as shown in Figure 4b). Because the timber elastic modulus perpendicular to the grain is much smaller than that of the other direction, the tenon is deformed due to the buried embedding and tends to slide on the contact surface between the tenon and the mortise, and the joint mainly relies on such deformation and on the frictional slip of the contact surface to provide the bending moment. As the external load continues to increase, the rotation also increases, the upper surface of the tenon and the mortise compress against each other, and similarly the upper surface of the tenon is deformed under compression perpendicular to the grain, accompanied by relative slip (as shown in Figure 4c). Under this condition, the joint mainly relies on this deformation and on the frictional slip of both the upper and lower surfaces of the tenon to provide the moment. Because the SMTJ/WP rotates about the wooden peg as the center and is symmetrical, its deformation pattern and stress state in forward rotation are the same as those in reverse rotation. Therefore, similar to SMTJs [11], the rotational moment generated by the SMTJ/WP under an external load is mainly provided by the extrusion force and sliding friction of the contact surface between the tenon and the mortise.

## 3. Rotational Moment–Rotation Relationship of the SMTJ/WP

### 3.1. Basic Assumptions

**Figure 5:**
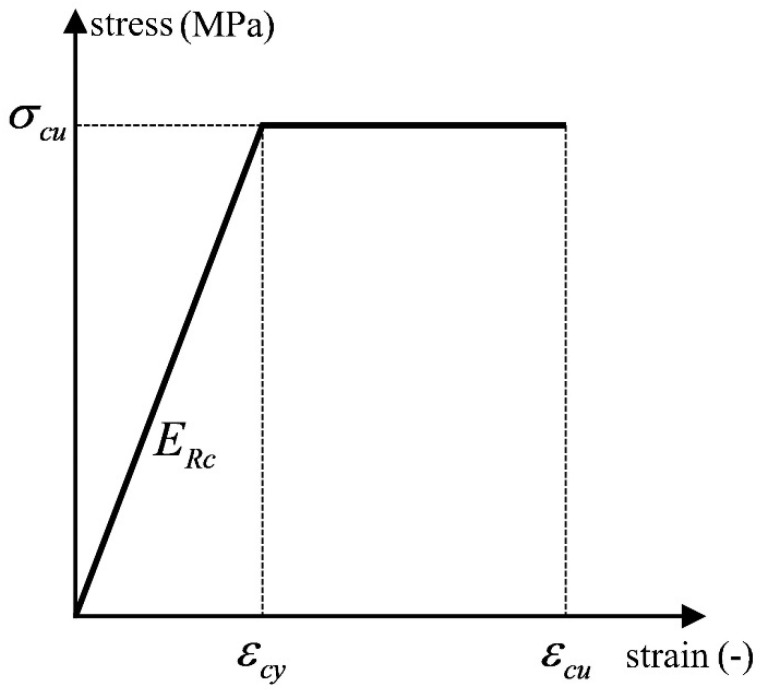
Double-linear constitutive model of wood perpendicular to the grain under compression. Note:$\;\sigma_{\mathrm{cu}}$ and $\varepsilon_{\mathrm{cy}}$ are the peak compressive stress and strain respectively; $\varepsilon_{\mathrm{cu}}$ is the ultimate compressive strain; $E_{\mathrm{Rc}}$ is the elastic modulus.

As seen from the stress mechanism analysis, the SMTJ/WP rotates with the wooden peg as the center under the external load and resists the external force through the extrusion deformation and frictional slip between the tenon and the mortise, and the stress states of the joint under forward and reverse loadings are the same. Based on the above analysis, the following assumptions are made to simplify the calculation:

1. The wooden peg is assumed to be a rigid body, and the extrusion deformation of the contact surface between the tenon and the wooden peg is neglected, i.e., the tenon rotates with the wooden peg as the center during the stressing process.
2. The influence of the interaction force between the peg and the peg hole of the beam on the bending moment is ignored. Since the pressure of the peg passes through the center of the peg, and the force arm from the friction in the tangential direction of the outer edge of the peg to the center of the peg is generally 5–15 mm, which is much smaller than other sizes, the contribution of the friction to the bending moment is small, and so the influence of the interaction force between the peg and the peg hole of the beam on the bending moment is not considered in the derivation of the theoretical model.
3. When the tenon and the mortise compress against each other, the mortise is compressed parallel to the grain, while the tenon is compressed perpendicular to the grain. According to Ref. [11] and the material property test, the compression elastic modulus of wood along the grain is ten times more than that of wood perpendicular to the grain. Therefore, the compression deformation of the mortise is ignored and it is assumed that only the tenon undergoes compression deformation.
4. Because the tenon is under uneven compression at multiple points, its bending deformation is relatively small, so its bending deformation is ignored based on the assumption of small deformation [11].
5. The longitudinal grain direction refers to the direction of the beam length and column height. During rotation, the main deformation occurs to the tenon, and the MTJ mainly depends on the compression force of wood perpendicular to the grain to provide the bending moment. Therefore, the derivation of a theoretical model is mainly based on the stress–strain relationship of wood perpendicular to the grain. As stress on the weakening segment slightly decreases, the constitutive relationship of wood under compression perpendicular to the grain is simplified to a bilinear constitutive model [17], as shown in Figure 5, and the stress–strain relationship is assumed to follow Hooke’s law [22].
6. The friction at the lateral contact surface between the tenon and the mortise is assumed to be 0. According to Reference [12], the lateral friction of the dovetail tenon contributes little to the moment of the joint, and the lateral restraint of the straight tenon is less than that of the dovetail tenon, so the influence of lateral friction can be neglected in the mechanical model.

### 3.2. Derivation of the Theoretical Moment Model for the SMTJ/WP

#### 3.2.1. Working States of the SMTJ/WP

Due to the presence of a gap between the tenon and the mortise, the MTJ may have a variety of working states. The moment provided by the extrusion between the tenon and the mortise varies among different working states. Therefore, the working state of the MTJ should be clarified before the derivation of the theoretical moment-rotation model of the joint.

**Figure 6:**
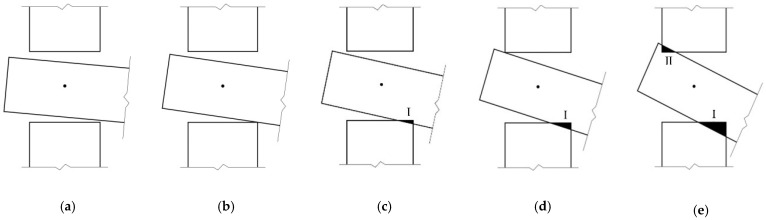
Working states under positive loading: (**a**) free rotation; (**b**) critical state Ι; (**c**) compression state I; (**d**) critical state II; (**e**) compression state II.

Some scholars [17] have studied the working state of penetrated mortise-tenon joints with gaps under forward and reverse loading and obtained the evaluation conditions for the working states of the MTJs. Referring to these research ideas, this paper gives the evaluation criteria of the working states for the SMTJ/WP with gaps. Taking the forward rotation as an example (assuming $\Delta_{g}^{s}>\Delta_{g}^{x}$, the same as below), in the initial rotation stage, the joint is in the free rotation state, as shown in Figure 6a, due to the existence of the joint gap. As the rotation increases, the lower surface of the tenon is in contact with the edge of the mortise, reaching critical state I, as shown in Figure 6b. When the bottom of the tenon is in close contact with the mortise, the bottom region I of the tenon and the mortise undergo significant compression (compression state I), as shown in Figure 6c. As the rotation continues, the upper surface of the tenon is in contact with the edge of the mortise, causing the joint to reach critical state II, as shown in Figure 6d. As the external load continues to increase, in addition to the compression between the bottom region I of the tenon and the mortise, the top region II of the tenon and the mortise also compress against each other, causing the joint to be in the compression state (compression state II), as shown in Figure 6e. Therefore, the working states of the joint are divided into free rotation, compression state I, and compression state II. In addition, Figure 6c,d show that the rotation at which region I enters the plastic state is not necessarily the same as the rotation corresponding to critical state II, so there may be two working states (elasticity and elasto-plasticity) for the wood in region Ι in compression state Ι. Figure 6e shows that under the same rotation, the compression area of the wood in region I (or region II) varies, and the rotation corresponding to the onset of the plastic state is also different. Therefore, compression state II can be divided into multiple working states.

#### 3.2.2. Determination of Working States

As one of the important parameters of the structural load-carrying capacity, the rotational moment of the SMTJ/WP is related not only to the geometrical dimensions and material parameters but also to the working state of the joint. Taking forward rotation as an example, there are different stress states, i.e., free rotation, compression state I and compression state II, of the tenon in the rotation process. In the compression state, the tenon undergoes a variety of working states. Therefore, the transition between working states should be fully considered in the analysis of the mechanical behavior of the SMTJ/WP.

Under cyclic loading, the working state of the joint is mainly determined by the critical rotations, where is the free rotation of the joint under forward loading, $\theta_{0}^{\prime }$ is the rotation corresponding to the critical state II, $\theta_{u}^{+}$ is the ultimate rotation, and $\theta_{Ie}$ and $\theta_{\mathrm{II}e}$ are respectively the critical rotation necessary for region Ι and region II to enter the plastic state. Because the rotation when the wood of the bottom region Ι of the tenon enters plasticity is not necessarily consistent with that when the upper surface of the tenon is only in contact with the mortise, it is necessary to compare the size between the two so as to correctly determine the working state of the joint in the process of rotation. If $\theta_{0}^{\prime }\leq \theta_{Ie}$, the tenon is only elastically compressed in compression state Ι; if $\theta_{0}^{\prime }>\theta_{Ie}$, the tenon appears elastic and elastoplastic compression occurs successively in extrusion state Ι. Therefore, the determination of the working states of the joint should be discussed in two cases:

1. $\theta_{0}^{\prime }\leq \theta_{Ie}$

When $0\leq \theta \leq \theta_{0}$, the joint is in free rotation; when $\theta_{0}<\theta \leq \theta_{0}^{\prime }$, the bottom region Ι of the tenon is in an elastic compression state; when $\theta_{0}^{\prime }<\theta \leq \theta_{Ie}$, the bottom region Ι and the top region II of the tenon are in elastic compression state; when $\theta_{Ie}<\theta \leq \theta_{\mathrm{II}e}$, the bottom region Ι of the tenon and the top region II of the tenon are in an elastoplastic extrusion state and elastic compression state, respectively; and when $\theta_{\mathrm{II}e}<\theta \leq \theta_{u}^{+}$, the bottom region Ι and the top region II of the tenon are in an elastoplastic compression state.

2. $\theta_{0}^{\prime }>\theta_{Ie}$

When $0\leq \theta \leq \theta_{0}$, the joint is in free rotation; when $\theta_{0}<\theta \leq \theta_{Ie}$, the bottom region Ι of the tenon is in an elastic compression state; when $\theta_{Ie}<\theta \leq \theta_{0}^{\prime }$, the bottom region Ι is in an elastoplastic compression state; when $\theta_{0}^{\prime }<\theta \leq \theta_{\mathrm{II}e}$, the bottom region Ι of the tenon and the top region II of the tenon are in an elastoplastic compression state and in an elastic compression state, respectively; when $\theta_{\mathrm{II}e}<\theta \leq \theta_{u}^{+}$, the bottom region Ι and the top region II are in an elastoplastic compression state.

#### 3.2.3. Theoretical Moment Model of the SMTJ/WP

Some scholars have deduced the theoretical models of several types of mortise–tenon joint with gaps [16,17,18,21], but the theoretical moment model of the SMTJ/WP involving a gap has not been proposed. Therefore, based on previous studies, this paper proposes a theoretical model of the SMTJ/WP, which is deduced as follows:

1. Free rotation

**Figure 7:**
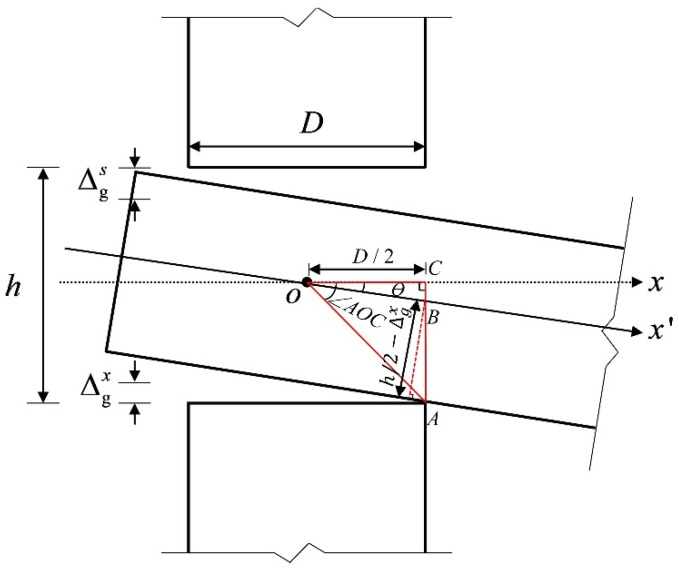
Free rotation.

At the initial loading, the tenon is in the free rotation state from the start of rotation until critical state I, as shown in Figure 7. Based on the assumptions and geometric relations, the free rotation of the joint can be obtained through the following analysis:

(1) $$\left|OA\right|=\sqrt{{\left(\frac{D}{2}\right)}^{2}+{\left(\frac{h}{2}\right)}^{2}}$$

(2) $$\left|OB\right|=\frac{D/2}{\cos\theta }$$

(3) $$\left|BA\right|=\frac{h/2-{∆}_{g}^{x}}{\cos\theta }$$

(4) $$∠AOC=\arctan\left(\frac{h/2}{D/2}\right)$$

(5) $${\left|BA\right|}^{2}={\left|OB\right|}^{2}+{\left|OA\right|}^{2}-2\left|OB\right|\times \left|OA\right|\cos(∠AOC-\theta )$$

where |OA|, |OB| and |BA| is the distance between two points, respectively; *Ɵ* is the rotation angle of the tenon; and *∠AOC* is the angle between two lines.

The free rotation $\theta_{0}$ is obtained by substituting Equations (1)–(4) into Equation (5) and inversely solving this using MATLAB.

2. Compression state

(a) Geometric relations

**Figure 8:**
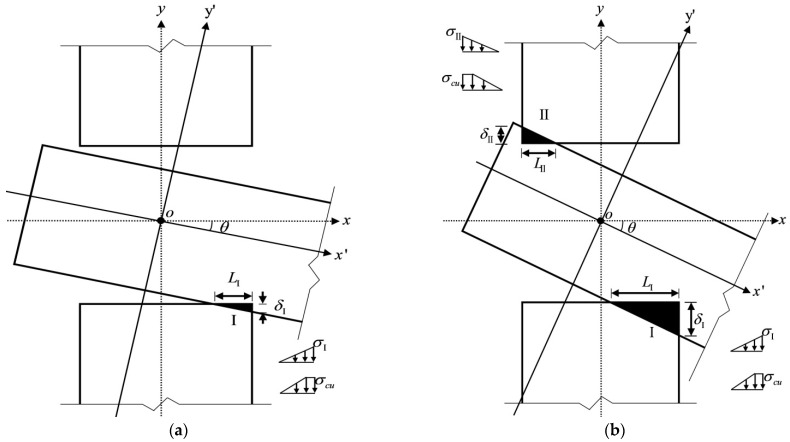
Stress state: (**a**) Compression state I and (**b**) Compression state Ⅱ.

When $\theta_{0}^{\prime }>\theta >\theta_{0}$, the lower part of the tenon and the mortise are in contact with each other, and the tenon is in the compression state I as shown in Figure 8a. Based on the geometric relations, the height of the compression region I in the lower part of the tenon can be obtained as follows:

(6) $$\delta_{I}=\frac{h/2-{∆}_{g}^{x}}{\cos\theta }+\frac{D}{2}\tan\theta -\frac{h}{2}$$

The length of the compression region I is

(7) $$L_{I}=\frac{\delta_{I}}{\tan\theta }$$

When $\theta >\theta_{0}^{\prime }$, the bottom of the tenon is tightly compressed with the mortise, and the upper part of the tenon and the mortise compress against each other, causing the tenon to be in the compression state II, as shown in Figure 8b. Similarly, the height and length of the compression region II are

(8) $$\delta_{\mathrm{II}}=\frac{h/2-{∆}_{g}^{s}}{\cos\theta }+\frac{D}{2}\tan\theta -\frac{h}{2}$$

(9) $$L_{\mathrm{II}}=\frac{\delta_{\mathrm{II}}}{\tan\theta }$$

(b) Physical conditions in the elastic state

According to the second assumption, when the wood is still in the elastic state, the compression stress in region I has not yet reached the ultimate compressive strength of the wood perpendicular to the grain, *σ*_cu_, and the stress is triangularly distributed, with the maximum stress given as follows:

(10) $$\sigma_{I}=\frac{\delta_{I}}{h-{∆}_{g}^{s}-{∆}_{g}^{x}}E_{Rc}$$

**Figure 9:**
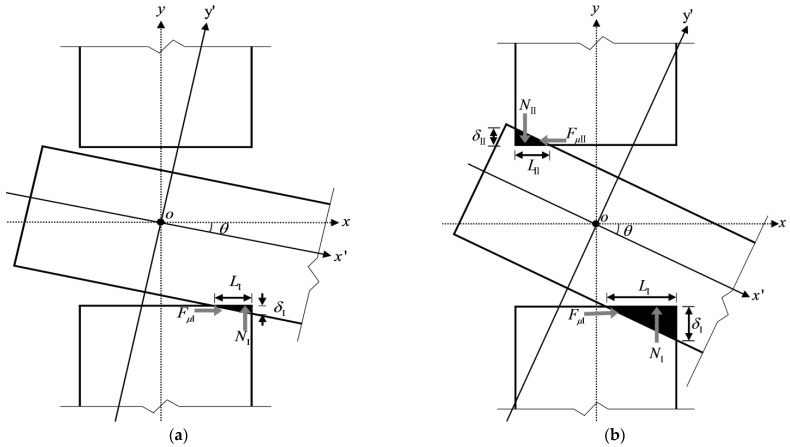
Force state: (**a**) Compression state Ι and (**b**) Compression state Ⅱ.

Then, the compression force and friction in region Ι in Figure 9a are

(11) $$N_{I}=\frac{1}{2}\sigma_{I}L_{I}b$$

(12) $$F_{\mu I}=\frac{1}{2}\sigma_{I}L_{I}b\mu$$

where *b* is the width of the tenon, and *μ* is the sliding friction coefficient between the wood perpendicular to the grain and the wood parallel to the grain (the same as below).

Similarly, the maximum compression stress of the wood in region II in the elastic compression state is

(13) $$\sigma_{\mathrm{II}}=\frac{\delta_{\mathrm{II}}}{h}E_{Rc}$$

Then, the compression force and friction in region II in Figure 9b are

(14) $$N_{\mathrm{II}}=\frac{1}{2}\sigma_{\mathrm{II}}L_{\mathrm{II}}b$$

(15) $$F_{\mu \mathrm{II}}=\frac{1}{2}\sigma_{\mathrm{II}}L_{\mathrm{II}}b\mu$$

(c) Conditions for determining elasto-plasticity

When the stress at the edge point reaches the ultimate compressive strength of the wood perpendicular to the grain, the tenon wood enters the plastic state; however, in the case of $\;{∆}_{g}^{s}>{∆}_{g}^{x}$, the compression areas on the upper and lower surfaces of the tenon are different, as are the moments in time when the wood enters the plastic state. Therefore, it is necessary to provide the conditions for determining that the wood on the upper and lower surfaces of the tenon has entered the plastic state.

(1) Condition for determining the plastic state in region I

When the stress at the edge point of region I reaches the ultimate compressive strength of the wood perpendicular to the grain, *σ*_cu_, the height and length of the compression zone at the edge point are obtained as follows:

(16) $$\delta_{Ie}=\frac{\sigma_{\mathrm{cu}}}{E_{Rc}}\left(h-{∆}_{g}^{s}-{∆}_{g}^{x}\right)$$

(17) $$L_{Ie}=\frac{\sigma_{\mathrm{cu}}\left(h-{∆}_{g}^{s}-{∆}_{g}^{x}\right)}{E_{Rc}\tan\theta }$$

According to the geometric relations, we have

(18) $${\left(\frac{h/2-{∆}_{g}^{x}}{\cos\theta }\right)}^{2}={\left(\frac{D/2}{\cos\theta }\right)}^{2}+{\left(\frac{D}{2}\right)}^{2}+{\left(\delta_{Ie}+\frac{h}{2}\right)}^{2}-2\frac{D/2}{\cos\theta }\sqrt{{\left(\frac{D}{2}\right)}^{2}+{\left(\delta_{Ie}+\frac{h}{2}\right)}^{2}}\cos\left[\arctan\left(\frac{\delta_{Ie}+\frac{h}{2}}{D/2}\right)-\theta \right]$$

The critical rotation for region Ι to enter the plastic state, $\theta_{Ie}$, is obtained by substituting Equation (16) into Equation (18) and inversely solving this by using MATLAB.

(2) Condition for determining the plastic state in region II

When the stress at the edge point of region II reaches *σ*_cu_, the height and length of the compression zone at the edge point are obtained as follows:

(19) $$\delta_{\mathrm{II}e}=\frac{\sigma_{\mathrm{cu}}}{E_{Rc}}\left(h-{∆}_{g}^{s}-{∆}_{g}^{x}\right)$$

(20) $$L_{\mathrm{II}e}=\frac{\sigma_{\mathrm{cu}}\left(h-{∆}_{g}^{s}-{∆}_{g}^{x}\right)}{E_{Rc}\tan\theta }$$

Similarly, the critical rotation for region II to enter the plastic state, $\theta_{\mathrm{II}e}$, can be obtained.

(d) Elastoplastic stage

(1) Elastoplasticity of region I

When $\theta >\theta_{Ie}$, region I enters the elastoplastic compression state. The compression height of the elastoplastic critical point and the compression length of the elastic section in the compression zone can be obtained from Equations (16) and (17), respectively. The compression height at the edge of the compression zone, $\delta_{I}$, and the total compression length of the compression zone, $L_{I}$, can be obtained from Equations (6) and (7), respectively. Then, the compression length of the plastic section is

(21) $$L_{Ip}=L_{I}-L_{Ie}$$

The compression force and friction of region I under elastoplastic compression are

(22) $$N_{Ip}=\frac{1}{2}\sigma_{\mathrm{cu}}L_{Ie}b+\sigma_{\mathrm{cu}}L_{Ip}b$$

(23) $$F_{\mu Ip}=\left(\frac{1}{2}\sigma_{\mathrm{cu}}L_{Ie}b+\sigma_{\mathrm{cu}}L_{Ip}b\right)\mu$$

(2) Elastoplasticity of region II

When $\theta >\theta_{\mathrm{II}e}$, region II enters the elastoplastic compression state. The compression height of the elastoplastic critical point and the compression length of the elastic section in the compression zone can be obtained from Equations (19) and (20), respectively. The compression height at the edge of the compression zone, $\delta_{\mathrm{II}}$, and the total compression length of the compression zone, $L_{\mathrm{II}}$, can be obtained from Equations (8) and (9), respectively. Then, the compression length of the plastic section is

(24) $$L_{Ip}=L_{I}-L_{Ie}$$

The compression force and friction of region II under elastoplastic compression are

(25) $$N_{\mathrm{II}p}=\frac{1}{2}\sigma_{\mathrm{cu}}L_{\mathrm{II}e}b+\sigma_{\mathrm{cu}}L_{\mathrm{II}p}b$$

(26) $$F_{\mu Ip}=\left(\frac{1}{2}\sigma_{\mathrm{cu}}L_{Ie}b+\sigma_{\mathrm{cu}}L_{Ip}b\right)\mu$$

(e) Theoretical moment-rotation model of the joint

According to the assumption, the moment caused by the friction between the side of the tenon and the sidewall of the mortise restraining the rotation of the tenon is ignored, and the moments in compression zones I and II of the tenon in the elastic compression stage are

(27) $$M_{Ie}=\frac{\sigma_{I}L_{I}b}{2}\left(\frac{D}{2}-\frac{1}{3}L_{I}\right)+\frac{h}{4}\sigma_{I}L_{I}b\mu$$

(28) $$M_{\mathrm{II}e}=\frac{\sigma_{\mathrm{II}}L_{\mathrm{II}}b}{2}\left(\frac{D}{2}-\frac{1}{3}L_{\mathrm{II}}\right)+\frac{h}{4}\sigma_{\mathrm{II}}L_{\mathrm{II}}b\mu$$

The moments of the tenon in compression zones I and II of the tenon in the elastoplastic compression stage are

(29) $$M_{Ip}=\frac{\sigma_{\mathrm{cu}}L_{Ie}b}{2}\left(\frac{D}{2}-L_{Ip}-\frac{1}{3}L_{Ie}\right)+\sigma_{\mathrm{cu}}L_{Ip}b\left(\frac{D}{2}-\frac{1}{2}L_{Ip}\right)+\frac{h}{2}\left(\frac{1}{2}\sigma_{\mathrm{cu}}L_{Ie}b+\sigma_{\mathrm{cu}}L_{Ip}b\right)\mu$$

(30) $$M_{\mathrm{II}p}=\frac{\sigma_{\mathrm{cu}}L_{\mathrm{II}e}b}{2}\left(\frac{D}{2}-L_{\mathrm{II}p}-\frac{1}{3}L_{\mathrm{II}e}\right)+\sigma_{\mathrm{cu}}L_{\mathrm{II}p}b\left(\frac{D}{2}-\frac{1}{2}L_{\mathrm{II}p}\right)+\frac{h}{2}\left(\frac{1}{2}\sigma_{\mathrm{cu}}L_{\mathrm{II}e}b+\sigma_{\mathrm{cu}}L_{\mathrm{II}p}b\right)\mu$$

The moment of the joint in each working state is

(1) When $\theta_{0}^{\prime }\leq \theta_{Ie}$

(31) $$M=\left\{ \begin{array}{l} 0,0\leq \theta <\theta_{0}\\ M_{Ie},\theta_{0}\leq \theta <\theta_{0}^{\prime }\\ M_{Ie}+M_{\mathrm{II}e},\theta_{0}^{\prime }\leq \theta <\theta_{Ie}\\ M_{Ip}+M_{\mathrm{II}e},\theta_{Ie}\leq \theta <\theta_{\mathrm{II}e}\\ M_{Ip}+M_{\mathrm{II}p},\theta_{\mathrm{II}e}\leq \theta <\theta_{u}^{+} \end{array}\right.$$

(2) When $\theta_{0}^{\prime }>\theta_{Ie}$

(32) $$M=\left\{ \begin{array}{l} 0,0\leq \theta <\theta_{0}\\ M_{Ie},\theta_{0}\leq \theta <\theta_{Ie}\\ M_{Ip},\theta_{Ie}\leq \theta <\theta_{0}^{\prime }\\ M_{Ip}+M_{\mathrm{II}e},\theta_{0}^{\prime }\leq \theta <\theta_{\mathrm{II}e}\\ M_{Ip}+M_{\mathrm{II}p},\theta_{\mathrm{II}e}\leq \theta <\theta_{u}^{+} \end{array}\right.$$

## 4. Experimental Verification

To verify the theoretical model equation, a low-cycle reversed loading test of the SMTJ/WP was carried out, and the theoretical model calculation results were compared with the experimental results.

### 4.1. Test Overview and Specimen Design

**Figure 10:**
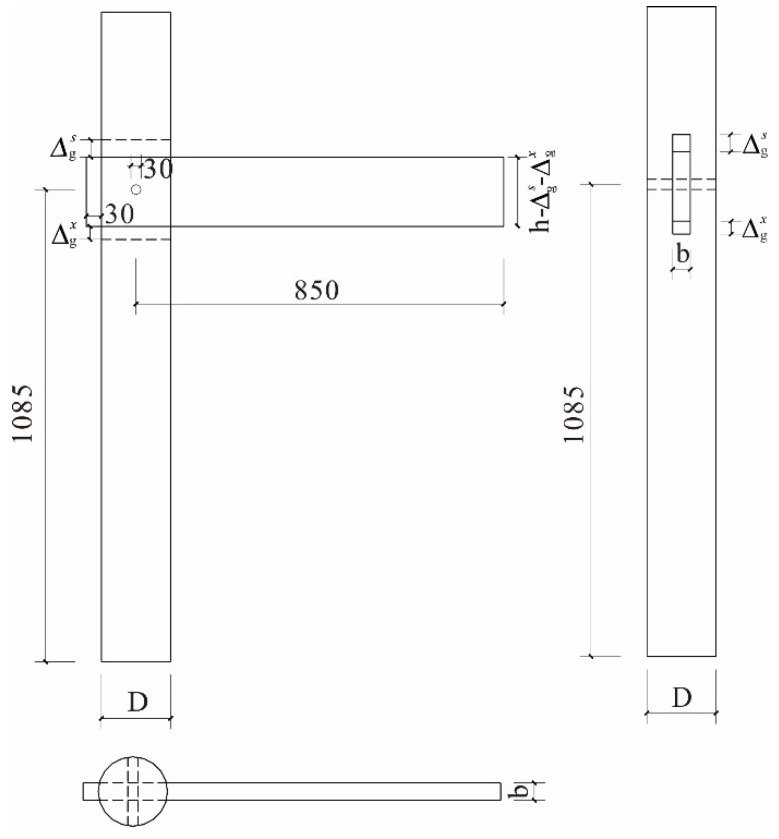
Specimen size and the details of the joint. Note: $h-{∆}_{g}^{s}-{∆}_{g}^{x}$ is the beam height after the radial shrinkage deformation perpendicular to the grain of the wood.

In 2019, some scholars from the school of architectural engineering of Kaili University measured the wooden structure dwellings in 10 villages in Southeast Guizhou, from which 10 dwellings were selected from each village for measurement, and so a total of 100 dwellings were measured. According to the rural survey results, the dimensions of the edge joints of the gable walls in most dwellings in southeastern Guizhou are in the following ranges: beam height, 150–250 mm; beam width, 30–60 mm; and column cross-section diameter, 160–250 mm. By comprehensively considering laboratory loading conditions and other factors, full-scale specimens were fabricated based on the rural survey results. A technical drawing of the tested specimens is shown in Figure 10. Based on the standard specimen (with geometric parameters of H = 1085 mm, L = 850 mm, h = 160 mm, and D = 180 mm) and considering the influence of the beam height and width parameters, three SMTJ specimens were fabricated, and the gap between the tenon and the mortise of each specimen was measured before the low-cycle reversed loading test was carried out. The specimens were named BS1–BS3, and their specific dimensions are given in Table 1.

### 4.2. Loading Protocol

**Figure 11:**
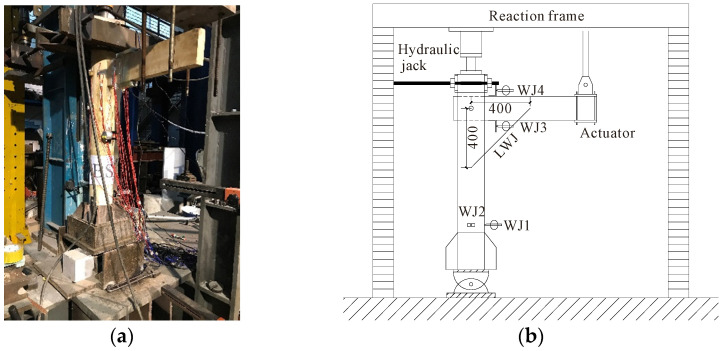
Loading equipment: (**a**) On-site loading and (**b**) Sketch.

The quasi-static loading method was adopted in this experiment, which was conducted at the State Key Laboratory of Subtropical Building Science at the South China University of Technology. The vertical load at the top of the column was applied by a hydraulic jack and remained constant during the entire loading process, and the cyclic loading at the beam end was applied by an MTS hydraulic actuator. The columns in traditional wooden houses generally stand directly on the foundation stone, and the column foot joint can be approximately treated as a hinge joint [23]. Therefore, in this experiment, the bottom of the column was fixed onto the ground by means of a one-way hinge support [24]. To avoid out-of-plane instability which may occur during the experiment, lateral supports were set outside the plane of the specimen [23]. The specific loading device is shown in Figure 11. The beam end load and the corresponding vertical displacement were measured by the load and displacement sensors of the MTS hydraulic actuator. Linear variable differential transducers (LVDTs) were placed on the inner side of the column above and below the beam end (WJ3 and WJ4, respectively, in Figure 11b), so that the rotation of the joint was measured by the ratio of the difference between the readings of the two displacement LVDTs to the vertical distance between the two LVDTs. A draw-wire displacement sensor (LWJ in Figure 11b) was arranged on the side of the column and at the bottom of the beam to calibrate the rotation measurement of the joint.

During the loading process, a vertical load of 11 kN was first applied at the top of the column, and then a vertical low-cycle reversed load was applied at the end of the beam using displacement control. When the control displacement was 2–8 mm, the control displacement increment was 2 mm at each level, with one cycle per level; when the control displacement was 16–80 mm, the increment was 8 mm at each level, with three cycles per level; thereafter, the increment was 16 mm at each level, with three cycles per level until the structure failed, when the loading was terminated.

### 4.3. Material Property Parameters

**Figure 12:**
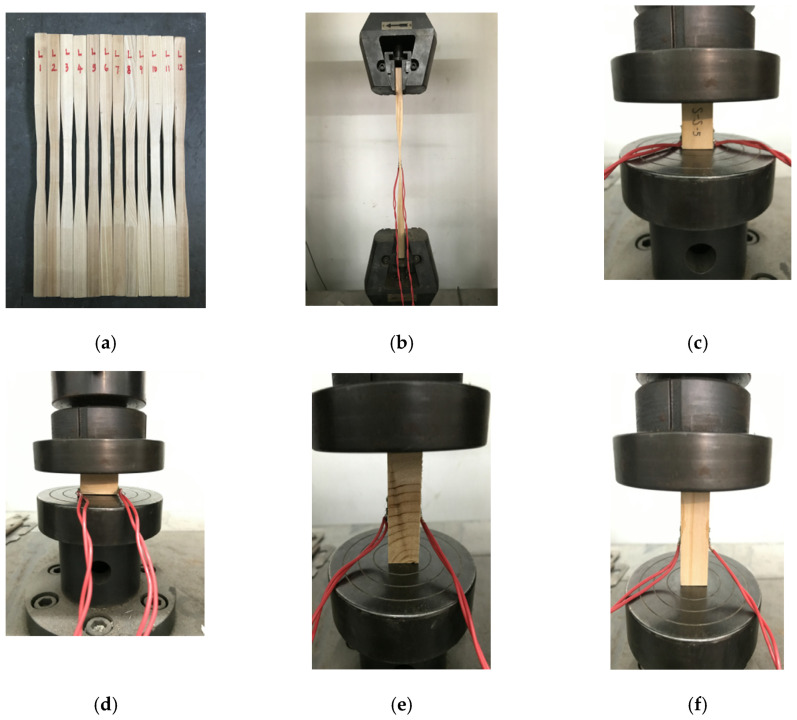
Parameter test of wood physical and mechanical properties: (**a**) tensile specimen; (**b**) tensile test; (**c**) compression specimen along the grain; (**d**) compression test along the radial; (**e**) the modulus of elasticity in compression along the radial; (**f**) the modulus of elasticity in compression along the grain.

The Chinese fir (*Cunninghamia lanceolata*) used in the test was taken from Kaili City, Southeast Guizhou. To understand the physical and mechanical properties of Chinese fir in this area, samples were taken from the same batch of wood as the joint experiment specimens and the physical and mechanical properties of the wood were tested, as shown in Figure 12.

**Figure 13:**
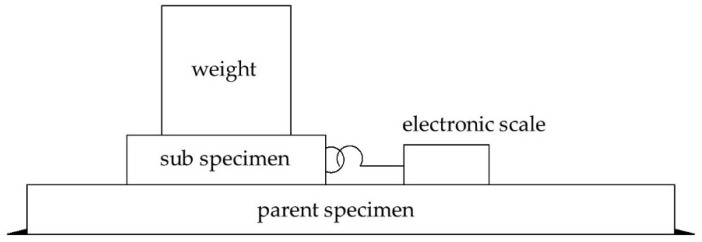
Loading diagram of the friction coefficient test. Note: the longitudinal grain direction of the parent specimen is horizontal; the longitudinal grain direction of the sub-specimen is vertical.

The material test was completed in the building materials laboratory and engineering mechanics center of the South China University of Technology. Different dimension and quantity of wood test pieces were selected according to different test indexes, among which the compressive strength parallel and perpendicular to the grain were respectively measured by 12 test pieces with a dimension of 30 mm × 20 mm × 20 mm and the modulus of elasticity in compression parallel and perpendicular to the grain were respectively measured by 12 pieces with a dimension of 60 mm × 20 mm × 20 mm. The test pieces were loaded according to the method for determination of each mechanical property. The collected data were analyzed and the results are shown in Table 2. Because the friction coefficient between the wood contact surface has a great influence on the energy dissipation capacity of the joint, the friction coefficient test of Chinese fir (Cunninghamia lanceolata) was carried out according to the method in Ref. [25]. When the mortise and tenon compress each other, the compression respectively occurs to the wood along the mortise grain and perpendicular to the tenon grain. Therefore, three groups of specimen were designed and manufactured to test the wood friction coefficient with different weights (2.469 kg, 4.868 kg, 9.713 kg, and 14.558 kg, respectively), and the test loading diagram is shown in Figure 13. The size of the parent specimen is 300 mm × 150 mm × 30 mm, and the direction along the grain is the length direction; the size of the sub-specimen is 100 mm × 100 mm × 50 mm, and the direction along the grain is the height direction. The electronic scale (range 0–50 kg) was used to pull the sub-specimen, and the reading of the electronic scale during the uniform sliding of the sub-specimen was used as the sliding friction of wood. To control the error, the friction force of each group of specimens under each level of weight was tested 5 times, and the average value was taken as the friction force under the weight. Then, the sliding friction coefficient under the weight is calculated according to formula (33). Finally, the average friction coefficient of three groups of specimens is taken as the friction coefficient of wood and the sliding friction coefficient *μ* is 0.42.

(33) $$\mu =\frac{F}{G_{1}+G_{2}}$$

where *F* is the sliding friction force; *G*_1_ is the weight; *G*_2_ is the self-weight of the sub-specimen.

### 4.4. Test Phenomenon and Failure Mode

**Figure 14:**
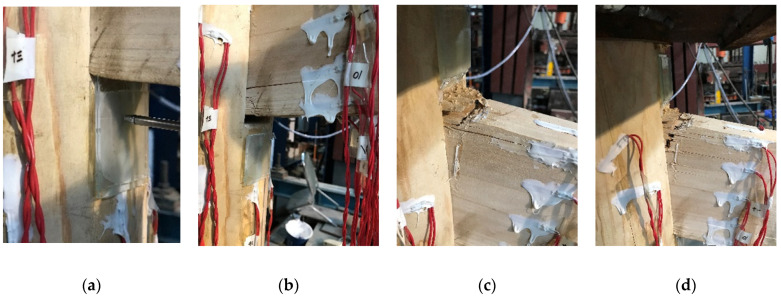
Experimental phenomena and failure modes of specimen BS1: (**a**) slight extrusion deformation; (**b**) cracking at the bottom of the tenon neck; (**c**) cracking at the top of the tenon neck; (**d**) tensile damage.

For specimen BS1, at the initial stage of loading, the tenon and the mortise were slightly deformed by mutual compression (Figure 14a). After unloading, there was no significant residual deformation of the tenon and the mortise, and the joint was basically in an elastic state. As the control displacement increased, a local fracture layer occurred at the top of the inner tenon neck of the column due to severe deformation under forward loading, the bottom fiber of the tenon neck suddenly pulled off and a wide transverse crack was formed under reverse loading (Figure 14b), while the top of the tenon and the edge of the inner mortise of the column extruded from each other to produce significant plastic deformation. As the load further increased, the fiber at the top of the tenon neck suddenly pulled off, and a clear transverse crack was generated (Figure 14c). As the loading continued, when the crack at the top of the tenon neck was large and extended to the wooden peg, tensile damage occurred (Figure 14d), and the joint failed.

**Figure 15:**
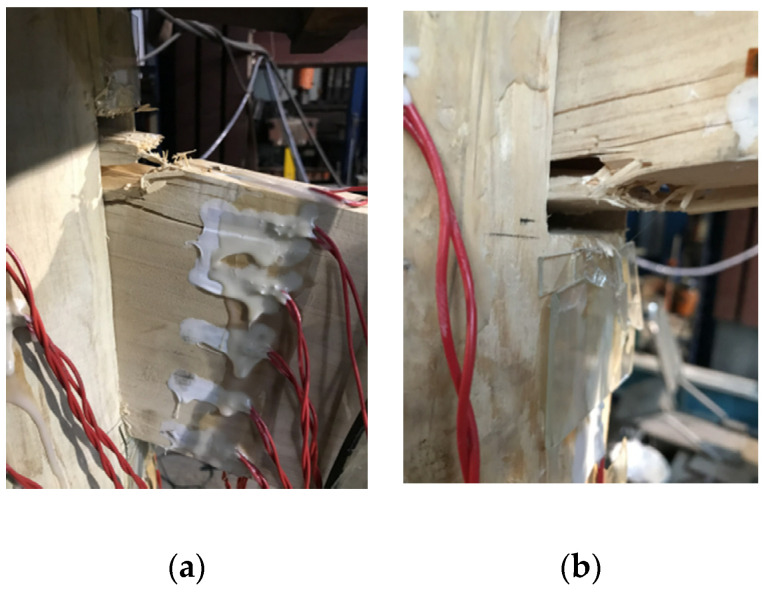
Failure modes of specimens BS2~BS3: (**a**) tensile crack failure at the top of the tenon neck; (**b**) fracture at the bottom of the tenon neck.

For specimens BS2–BS3, the test phenomenon at the beginning and middle stage of loading was similar to that of specimen BS1, but the final failure mode of each specimen is different. Transverse cracks were generated at the upper end of the beam tenon of specimen BS2 under tension, resulting in the ultimate tensile crack failure along the grain (Figure 15a). The lower part of the beam tenon of specimen BS3 was extruded to generate transverse cracks, which eventually led to the fracture of the specimen (Figure 15b). After failure, the test load decreased rapidly, the specimen lost the ability to continue bearing, and the test loading was terminated. Although the cracks in specimens BS2 and BS3 did not appear at the same time and location, they both extended finally to the wooden peg, with a failure mode similar to that of BS1.

### 4.5. Theoretical Model Validation

**Figure 16:**
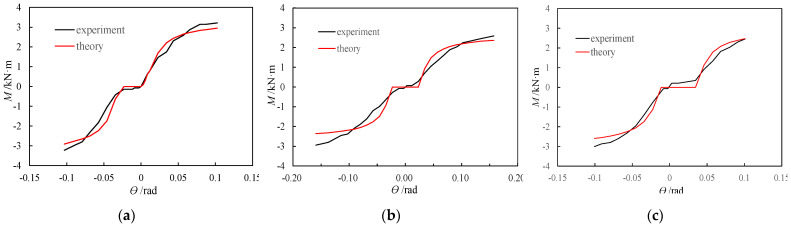
Comparison of experimental versus theoretical moment-rotation curves: (**a**) BS1; (**b**) BS2; (**c**) BS3.

The average values given in Table 2 are substituted into the formula of the theoretical moment model to obtain the theoretical moment-rotation relationship curve, and then the moment–rotation backbone curves obtained from the theoretical calculations and tests are plotted in Figure 16. The analysis shows that there is a difference between the theoretical curve and the test curve at the initial stage of loading. This result is mainly because, in the actual test environment, there was friction between the side of the tenon along the width of the tenon cross-section and the side of the mortise; because there was a small amount of moment due to friction, the test moment was small (but not zero) in the free rotation stage, while such friction was ignored in the theoretical calculation, so the theoretical moment was zero. In the stage of mutual extrusion of the tenon and the mortise when the tenon rotation angle is larger than the free rotation angle, the experimental curve differs from the theoretical curve. The difference arises from the large dispersion of wood as well as the use of a bilinear model for the constitutive relationship of wood under compression perpendicular to the grain and without the consideration of the change in the wood strength with plasticity in the theoretical calculation. In addition, for BS1, the self-weight of the beam caused a slight rotation of the beam end so that the bottom of the tenon was already in contact with the edge of the inner mortise of the column before loading. Therefore, the curve contains no slip section for loading in the forward direction. During the reverse loading, there was a gap (approximately 2 mm) between the bottom of the tenon and the edge of the outer mortise of the column, so the curve had a slip section, and the moment increased significantly only after the bottom of the tenon was in contact with the edge of the outer mortise of the column and compression occurred. For BS3, the gap between the top of the tenon and the edge of the inner mortise of the column was reduced by approximately 1 mm during the installation of the specimen, so the gap between the bottom of the tenon and the edge of the inner mortise of the column increased to approximately 3 mm; therefore, in the curve, the slip section during the forward loading is larger than that during the reverse loading. The comparison of the test data and the theoretical calculation results (as shown in Table 3) showed that the average of *M*_yc/_*M*_ye_ is 0.970, the standard deviation amounts to 0.070, and variable coefficient equals to 0.072. These characteristic indices of *M*_uc_/*M*_ue_ are 0.897, 0.042 and 0.047, respectively. The values of the characteristic points shown in the table are the mean values of positive and negative loads. It can be seen from Figure 15 and Table 3 that, although the calculation and experimental results are not same, the overall change trends of the two curves are roughly similar, which reflects the mechanical properties of the SMTJ/WP during the rotation process. In addition, from the comparison between the experimental moment-rotation curve and the theoretical moment-rotation curve, it can be seen that the basic assumptions adopted by the theoretical model are reasonable, and the model can be used for parametric analysis on this basis.

## 5. Analysis of the Influence of the Parameters

According to the theoretical moment-rotation calculation equation, the parameters affecting the moment-rotation relationship of the SMTJ/WP include the beam height *h*, the column diameter *D*, the beam width *b*, the sliding friction coefficient *μ* between the wood parallel to the grain and the wood perpendicular to the grain, and the gap ${∆}_{g}^{s}$ between the tenon and the mortise (assuming ${∆}_{g}^{x}={∆}_{g}^{s}$). These influencing factors are analyzed separately below.

### 5.1. Influence of Beam Height

**Figure 17:**
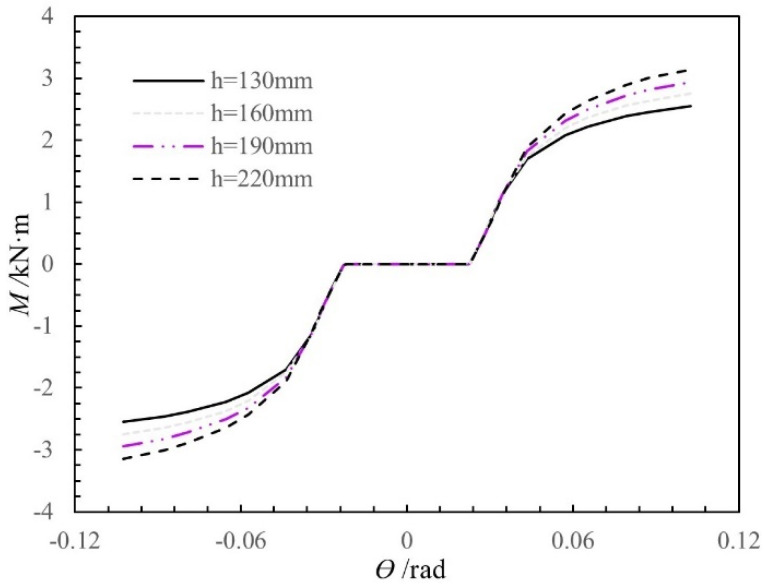
Moment-rotation curves of SMTJ/WP for different depths of the tenon.

In the analysis, the standard model has the following parameters: a beam height of 160 mm, a beam width of 50 mm, a column diameter of 180 mm, and a gap of 2 mm between the tenon and the mortise. Figure 17 shows the moment-rotation curves calculated using the theoretical moment-rotation model for different beam heights (130 mm, 160 mm, 190 mm, and 220 mm), with the other influencing factors held constant. Before the joint yields, changing the beam height does not significantly influence the rotational stiffness and the moment; however, after the joint yields, the rotational stiffness and the moment of the joint both increase as the beam height increases. The influence of the beam height factor on the moment and stiffness of the joint before and after yielding is inconsistent because, as the beam height increases, the increases in the compression height and the compression length calculated from Equations (6) and (7), respectively, are greater after joint yielding than before joint yielding. As a result, the increase in the moment is also greater after joint yielding than before joint yielding, so the rotational stiffness and the moment increase significantly after joint yielding as the beam height increases.

### 5.2. Influence of Column Diameter

**Figure 18:**
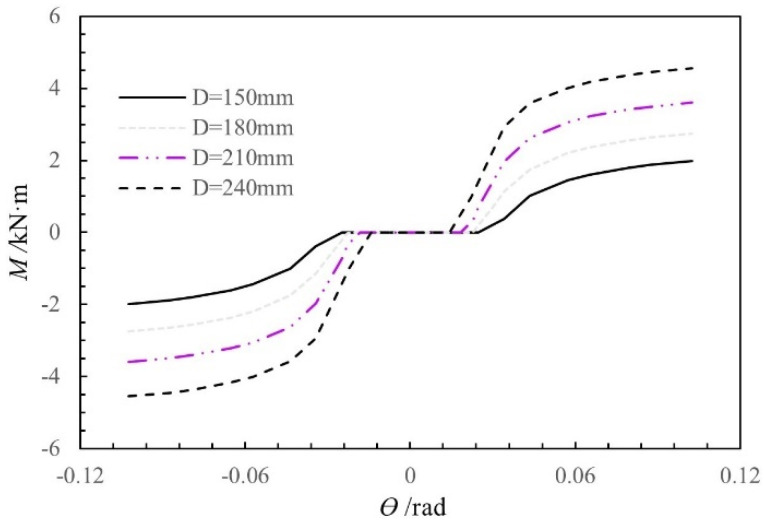
Moment-rotation curves of SMTJ/WP for different diameters of the column.

The moment-rotation curves were calculated using the theoretical moment-rotation model for different column diameters (150 mm, 180 mm, 210 mm, and 240 mm), with the other influencing factors held constant. As shown in Figure 18, as the column diameter increases in the range of 150–240 mm, the initial stiffness and moment of the SMTJ/WP increase, while the free rotation gradually decreases.

### 5.3. Influence of Beam Width

**Figure 19:**
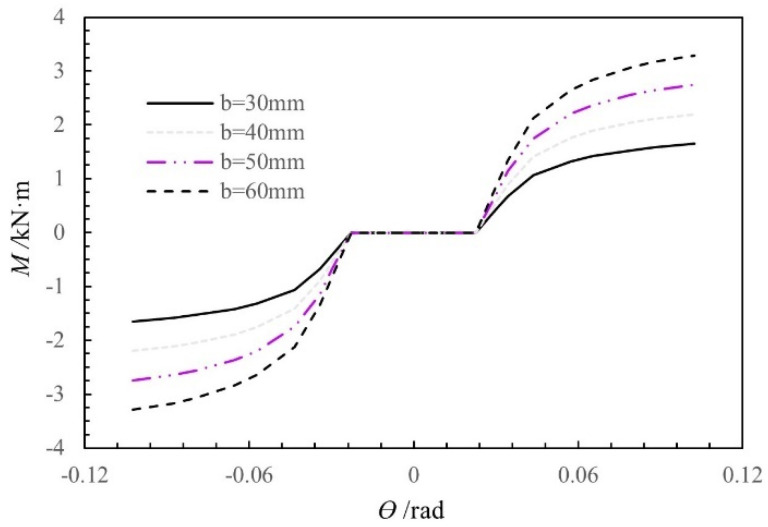
Moment-rotation curves of the SMTJ/WP at different widths of the tenon.

Figure 19 shows the moment-rotation curves obtained using the theoretical moment-rotation model for different beam widths (30 mm, 40 mm, 50 mm, and 60 mm), with the other influencing factors held constant. The rotational stiffness and moment of the SMTJ/WP increase as the beam width increases from 30 mm to 60 mm because an increase in the beam width causes an increase in the extrusion contact area, which leads to an increase in the resultant extrusion force and friction, thereby causing the rotational stiffness and moment of the joint to increase as the beam width increases.

### 5.4. Influence of Sliding Friction Coefficient μ

**Figure 20:**
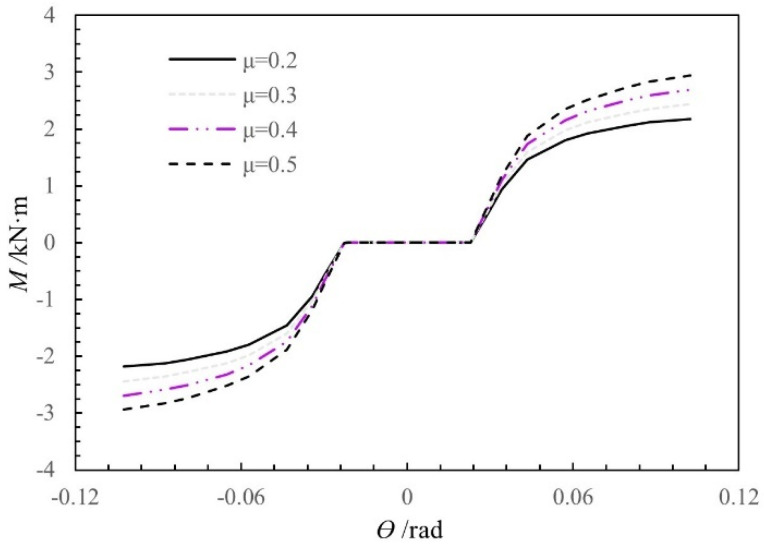
Moment-rotation curves of SMTJ/WP at different friction coefficients of wood.

According to the Reference [26], the sliding friction coefficient between wood, *μ*, is in the range of 0.10–0.65. Based on the standard model, the influence of *μ* on the moment-rotation relationship of the SMTJ/WP was analyzed by considering different *μ* values (0.2, 0.3, 0.4, and 0.5), with the results shown in Figure 20. Overall, changing the friction coefficient has a large influence on the moment of the joint. Before and after the joint yields, both the rotational stiffness and the moment increase as the friction coefficient increases. Locally, the increases in the rotational stiffness and moment are larger after the joint yields than before. The increases in the rotational stiffness and moment before and after the joint yields are inconsistent. because the compression between the tenon and the mortise of the joint is closer after the joint yields than before, and the compression contact area between the tenon and the mortise increases continuously, thereby significantly increasing the friction.

### 5.5. Influence of the Gap between the Tenon and the Mortise

**Figure 21:**
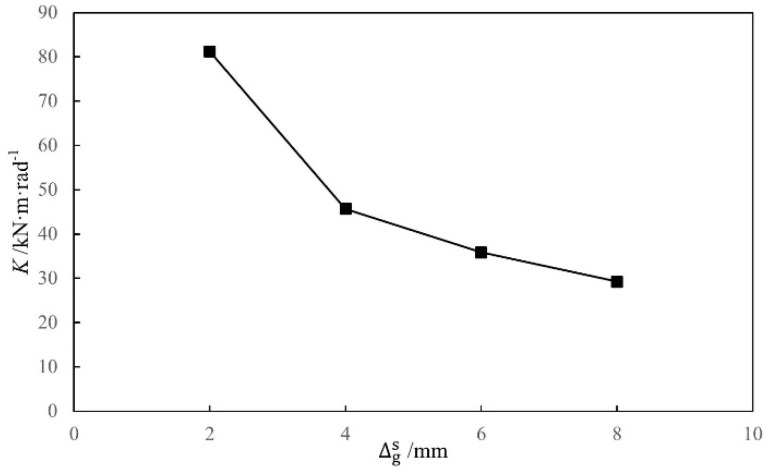
Moment-rotation curves of SMTJ/WP at different widths of the gap.

The gap between the tenon and the mortise, ${∆}_{g}^{s}$ (assuming ${∆}_{g}^{x}={∆}_{g}^{s}$), was changed to analyze its influence on the initial rotational stiffness of the SMTJ. The gap in the MTJ is assumed to be caused primarily by the shrinkage deformation of the tenon wood in the radial direction perpendicular to the grain, and the radial shrinkage deformation of the wood is generally 3–6% of the height of the tenon [21]. Therefore, based on the cross-sectional height of the tenon of the standard model, the gap ${∆}_{g}^{s}$ was used as a parameter for analysis in this study. The influence of the gap on the initial rotational stiffness of the joint was investigated by using different ${∆}_{g}^{s}$ values (2 mm, 4 mm, 6 mm, and 8 mm), with the influence curves shown in Figure 21. Clearly, as the gap between the tenon and the mortise increases, the initial rotational stiffness of the joint decreases because the area of the embedment zone between the tenon and the mortise decreases as the gap increases, resulting in a decrease in the rotational moment of the joint, which in turn causes the initial rotational stiffness of the joint to decrease as the gap ${∆}_{g}^{s}$ increases.

## 6. Conclusions

This study theoretically analyzed the mechanical performance of an SMTJ/WP involving gaps. The conclusions are as follows:

(1) The mechanical behavior of a SMTJ/WP involving a gap is closely related to the working states of the joint. According to the comparison between the rotation angle corresponding to the critical state II and the critical rotation angle for region Ι to enter the plastic state, the judgment criteria of the working state of the joint in two cases are obtained. For the first case, the joint has only one working state in compression state Ι; that is, the elastic compression state of region Ι. For the second case, two working states in compression state Ι, namely, the elastic compression and elastoplastic compression states, occur in region Ι successively.
(2) The theoretical moment–rotation model of the SMTJ/WP involving a gap is proposed for different working states. Compared with the experimental results, the theoretical calculation results are generally consistent, indicating the validity of the theoretical calculation method for the SMTJ/WP.
(3) According to the theoretical model, the analysis of the factors affecting the *M*-*Ɵ* relationship of the SMTJ/WP showed that both the rotational stiffness and the moment of the joint tend to increase as the beam width increases. Before the joint yields, the change in the beam height has no significant influence on the moment and rotational stiffness; after the joint yields, both the rotational stiffness and the moment increase as the beam height increases.
(4) Furthermore, the derived model indicates that the increase in the column diameter leads to an increase in both the initial stiffness and the moment of the joint and a decrease in its free rotation, while the gap between the tenon and the mortise has the opposite influence on the initial stiffness, moment, and free rotation. The rotational stiffness and moment of the SMTJ/WP increase as the sliding friction coefficient increases, but the increase is larger after the joint yields than before.

## Figures and Tables

**Table 1:** The joint parameters.

Specimen	*h*/mm	*b*/mm	${∆}_{g}^{s}/\mathrm{mm}$	${∆}_{g}^{x}/\mathrm{mm}$
BS1	160	50	2	1
BS2	160	40	2	2
BS3	130	50	2	2

**Table 2:** Physical and mechanical properties of Chinese fir (Cunninghamia lanceolata).

Experimental Items	Sample Size	Average Value/MPa	Standard Deviation/MPa	Variation Coefficient %	Moisture Content %
Compressive strength along the grain	30 mm × 20 mm × 20 mm	31.13	0.91	2.93	11.3
Compressive strength perpendicular to the grain	30 mm × 20 mm × 20 mm	4.33	0.69	15.83	12.4
Tensile strength along the grain	370 mm × 20 mm × 15 mm (The middle section size is 15 mm × 5 mm)	75.27	6.97	9.26	11.7
Elastic modulus along the radial	60 mm × 20 mm × 20 mm	1073.96	109.53	10.2	12.2
Elastic modulus along the grain	60 mm × 20 mm × 20 mm	11430.84	805.82	7.05	11.9

**Table 3:** Comparison analysis.

Specimen	*M*_ye_/KN·m	*M*_yc_/KN·m	*M*_yc_ */M*_ye_	*M*_ue_/KN·m	*M*_uc_/KN·m	*M*_uc_ */M*_ue_
BS1	1.65	1.72	1.04	3.22	2.93	0.91
BS2	1.02	0.92	0.90	2.77	2.36	0.85
BS3	1.16	1.13	0.97	2.73	2.53	0.93

Note: *M*_y_ and *M*_u_ represent yield moment and the ultimate moment, respectively; the subscripts “e” and “c” denote the experimental values and the calculated values, respectively.

## Data Availability

Data is contained within the article.
